# Microscopic Inspection of the Retina

**Published:** 1857-04

**Authors:** 


*Microscopic Inspection of the Retina.*—The following, which we copy from the *Medical News*, Philadelphia, furnishes the first explanation we have seen of the circumstances which a few weeks since furnished a subjec for wide spread newspaper comment. We wonder if the *London Lancet* will be willing to settle for the ox, now the ownership of the bull is discovered:

[Some of our readers may have noticed in a recent number of the London Lancet, some flippant reflections upon American physicians, founded upon a statement which has been going the rounds of the daily press, in regard to a method of detecting a murderer by examining the eyes of the murdered man, upon whose retina it was said the image of the murderer would be painted. The following letter somewhat amusingly turns the table upon the critic:]

*Your Bull and my Ox.*—To the Editor of the *Medical News.*—SIR: I observe in a recent number of the *London Lancet*, a severe censure upon American medical jurisprudence, based upon a statement published by two persons, at Auburn, in New York, of their microscopic inspection of the eye of a murdered man.

This excels the Arrowsmith absurdity, on the part of our well-informed English cousins. The facts in this matter are the following: The coroner, at Auburn, Mr. Sittzer, though not learned, is a man of practical discernment and good sense. He did not direct this eye exploration to be made, nor did he receive any testimony on that point. The investigation under notice was a volunteer affair, assented to by the friends of the murdered man, and was conducted by Dr. Bellamy, an *Englishman*, who had come to this country to practice medicine and surgery under an English license. He was assisted by a young homœopathic practitioner.

As the coroner rejected this volunteer information, Dr. Bellamy, doubtless, that it might not be lost to the world, with true national pride, had it published over his own signature in a political newspaper.

Respectfully yours,

Auburn, N. Y., Feb. 1857. THEO. DIMON, M. D.

